# A Molecular Imaging Strategy for In Vivo Monitoring of Vascular Remodeling in a Porcine Model of Femoral Artery Overdilation

**DOI:** 10.1007/s10439-026-04141-6

**Published:** 2026-04-13

**Authors:** Mahboubeh Nabavinia, Anjana Jayaraman, Eleanor T. Rimmerman, Kumudha Narayana Musini, Corrin R. Mansfield, Katherine E. Mevis, Michael R. Go, Mitchel R. Stacy

**Affiliations:** 1 Center for Regenerative Medicine, The Research Institute at Nationwide Children’s Hospital, Columbus, OH USA; 2 Interdisciplinary Biophysics Graduate Program, The Ohio State University, Columbus, OH USA; 3 Division of Vascular Diseases and Surgery, Department of Surgery, The Ohio State University College of Medicine, Columbus, OH USA; 4 Division of Vascular Diseases and Surgery, Center for Regenerative Medicine, Research Institute at Nationwide Children’s Hospital, The Ohio State University College of Medicine, 575 Children’s Crossroad, Room WB4133, Columbus, OH 43215 USA

**Keywords:** Positron emission tomography, Imaging, Fluorodeoxyglucose, Peripheral artery disease, Vascular, Angioplasty

## Abstract

**Purpose:**

Clinical use of endovascular interventions has increased in recent decades for treatment of peripheral artery disease (PAD); however, molecular imaging paradigms for monitoring inflammatory responses or adverse remodeling in targeted arteries have not been validated. Therefore, this study tested the utility of positron emission tomography (PET) imaging with fluorine-18 (^18^F)-fluorodeoxyglucose (FDG) for in vivo detection of peripheral angioplasty-induced arterial remodeling.

**Methods:**

Yorkshire pigs (*n* = 8) underwent fluoroscopy-guided overdilation of the right femoral artery using a balloon catheter inflated 1.25–1.85X the arterial diameter. ^18^F-FDG PET/CT imaging was performed 14 days post-angioplasty. Injured and control arteries were harvested immediately after imaging and sectioned for gamma counting (^18^F-FDG uptake) and stained with hematoxylin and eosin (H&E), alpha-smooth muscle actin (α-SMA), and CD64 for evaluation of arterial thickness, smooth muscle cell area, and macrophage infiltration.

**Results:**

In vivo PET/CT imaging detected a significant increase in ^18^F-FDG uptake in injured versus control femoral arteries 14 days post-angioplasty (*p* = 0.008) that was confirmed by ex vivo gamma counting of vessels (*p* = 0.02). Ex vivo gamma counting of ^18^F-FDG uptake demonstrated a significant association with in vivo ^18^F-FDG uptake measured by PET/CT imaging (*r* = 0.92; *p* = 0.004). Image analysis for H&E, α-SMA, and CD64 revealed significant relative increases in vessel wall thickness (*p* < 0.0001), smooth muscle cell area (*p* = 0.0001), and CD64 area (*p* = 0.04) that were each significantly associated with ex vivo measures of ^18^F-FDG uptake.

**Conclusion:**

^18^F-FDG PET/CT quantifies remodeling characteristics associated with angioplasty-induced injury, thereby providing a potential in vivo paradigm for monitoring and testing of emerging devices for PAD treatment.

## Introduction

Peripheral artery disease (PAD) is an atherosclerotic disease of the lower extremities that affects an estimated 230 million people worldwide, significantly impairs quality of life and functional capacity, and is associated with elevated risk for limb loss [1]. Adequate limb blood flow and skeletal muscle perfusion are critical for symptom management, maintenance of functional status, and limb preservation in patients with PAD [2]. The clinical use of endovascular-guided revascularization has increased in recent decades for the treatment of limb ischemia in severe stages of PAD [3–5]; however, development of restenosis and the need for re-intervention remains highly prevalent despite the continued emergence of drug-coated and drug-eluting endovascular devices that are intended to have anti-proliferative effects [6]. Standard evaluation of treatment response to peripheral interventions largely consists of intra-operative evaluation of arterial patency (via angiography) and post-operative monitoring of peripheral hemodynamics (e.g., ankle-brachial index) [7], but a non-invasive imaging approach does not exist for quantifying the in vivo physiological responses to endovascular interventions within target lesions/vessels that may be associated with adverse arterial remodeling and potentially predict risk of restenosis after intervention.

Fluorine-18 (^18^F)-fluorodeoxyglucose (FDG) positron emission tomography (PET)/computed tomography (CT) imaging is a well-established approach for non-invasively quantifying arterial inflammation in the setting of coronary [8] and peripheral [9] atherosclerosis and has demonstrated potential for detecting vascular smooth muscle cells and their metabolic activity in pre-clinical models of atherosclerosis [10, 11]. Additionally, arterial retention of ^18^F-FDG on PET/CT images prior to peripheral angioplasty has shown prognostic value for predicting subsequent 12 month incidence of restenosis in patients with PAD [12]. However, to our knowledge, ^18^F-FDG PET/CT imaging has not specifically been used to quantify the inflammatory or vascular remodeling responses associated with peripheral endovascular interventions. Therefore, the present study sought to test and validate ^18^F-FDG PET/CT imaging as a non-invasive biomarker of peripheral balloon angioplasty-induced arterial injury and evaluate the relationship between arterial retention of ^18^F-FDG and histological measures of arterial remodeling. We hypothesized that ^18^F-FDG PET/CT imaging could detect both the physiological and morphological remodeling induced by balloon overdilation in a porcine model and that in vivo arterial retention of ^18^F-FDG would be associated with characteristics of arterial remodeling.

## Materials and Methods

### Animal Surgical Preparation and Procedures

Experimental protocols were approved by the Institutional Animal Care and Use Committee of Nationwide Children’s Hospital and were in compliance with the Association for Assessment and Accreditation of Laboratory Animal Care International policies. All procedures performed in animals also conformed to the National Institutes of Health Guide for the Care and Use of Laboratory Animals. The use of radioisotopes and all PET/CT imaging procedures were approved by the Radiation Safety Committee of Nationwide Children’s Hospital.

Eight female Yorkshire pigs (23.4 ± 4.2 kg) approximately 8 weeks of age underwent percutaneous transluminal angioplasty of the right femoral artery to induce vascular injury. Animals were fed and maintained on a standard chow diet for the duration of the study. Prior to surgery, animals were fasted for a minimum of 8 h. Animals were then sedated by intramuscular administration of ketamine (15–20 mg/kg) and xylazine (1–2 mg/kg), followed by intubation and mechanical ventilation with 100% oxygen (VentiPAC, Smiths Medical International Ltd., Luton, UK). For maintenance of anesthesia during surgery, animals were intravenously administered propofol at a continuous rate of 10–30 mg/kg/hour through an ear vein catheter. Depth of anesthesia was monitored every 15 min by evaluating blink reflex and jaw tone. Blood pressure, heart rate, oxygen saturation, and body temperature were continuously monitored for the duration of the surgery (IntelliVue MP30; Philips Healthcare, Andover, MA).

**Fig. 1:**
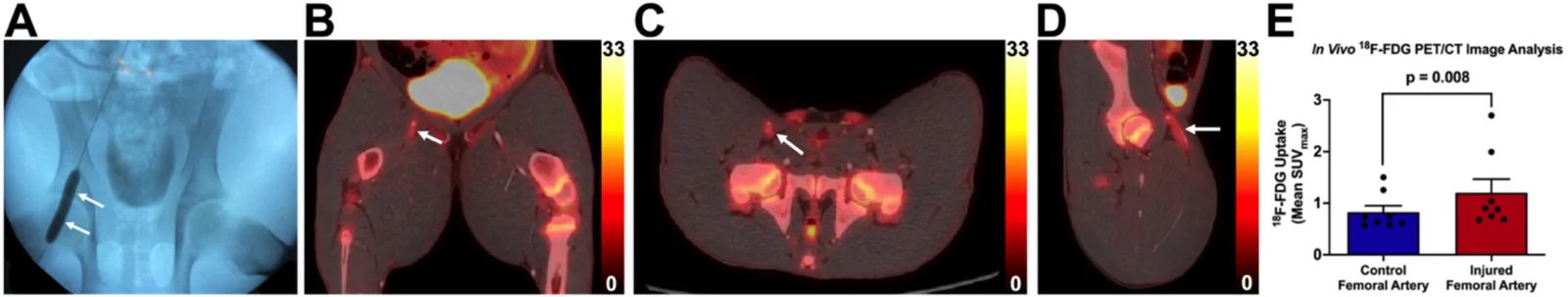
Porcine model of femoral artery balloon overdilation-induced injury and lower extremity ^18^F-FDG PET/CT imaging. **A** Representative fluoroscopy imaging of unilateral femoral artery balloon angioplasty performed in a porcine model under fluoroscopic guidance. Representative **B** coronal, **C** axial, and **D** sagittal views of lower extremity ^18^F-FDG PET/CT imaging 2 weeks after balloon-induced injury, demonstrating increased ^18^F-FDG uptake localized to the right femoral artery (denoted by white arrows). **E** Quantitative analysis of in vivo PET/CT images confirmed significantly higher ^18^F-FDG uptake in the injured versus control femoral artery, *n* = 8 animals. Data are shown as means ± SEM

To induce unilateral femoral artery injury via balloon angioplasty, the carotid artery was first accessed by percutaneous puncture, and a 5-F polyethylene introducer sheath (PINNACLE®, Terumo, Somerset, NJ, USA) was placed. 100 units of heparin per kilogram of body weight were intravenously administered prior to the insertion of wires. An 0.035” J-tip guidewire (Boston Scientific, Marlborough, MA, USA) was then positioned in the distal femoral artery under fluoroscopy guidance (OEC 9900 Elite C-Arm, GE Healthcare, Chicago, IL, USA), a 4-F angiocatheter (Mongoose, Pediavascular, Chagrin Falls, OH, USA) was inserted over the guidewire to the level of the abdominal aorta, the wire was removed, and an angiographic roadmap of the lower extremities was subsequently acquired (Fig. 1A). The guidewire was re-inserted and the angiocatheter was then exchanged for a 5-F over-the-wire balloon catheter (7 × 40 mm, Mustang Dilatation Catheter, Boston Scientific, Marlborough, MA, USA), which was inflated once for 3 min using a balloon-to-artery ratio ranging from ~ 1.25 to 1.85 to induce varying degrees of injury to the right femoral artery. The balloon was then deflated, the catheter and wire were removed, and protamine was intravenously administered (1 mg per 100 units of heparin administered) prior to removal of the carotid artery sheath to limit bleeding. Details of the femoral artery and balloon characteristics are outlined in Table 1.

**Table 1:** Femoral artery and balloon characteristics

Animal ID	Control artery diameter (mm)	Injured artery diameter (mm)	Balloon inflated pressure (atm)	Balloon diameter (mm)	Balloon-to-artery ratio
1	4.44	4.85	8	6.96	1.57
2	5.56	5.61	6	6.9	1.24
3	3.78	5.56	10	7.02	1.57
4	4.95	6.8	10	7.02	1.86
5	4.77	4.95	11	7.07	1.48
6	4.54	4.92	10	7.02	1.55
7	4.84	4.85	11	7.07	1.46
8	3.80	4.27	6	6.9	1.82
Mean	4.58	5.23	9	7.0	1.55
St. Dev.	0.6	0.8	2.1	0.1	0.2

### ^18^F-FDG PET/CT Imaging Protocol

Animals were fasted prior to all imaging procedures for a minimum of 8 h. Propofol was intravenously infused at 10–30 mg/kg/h through an ear vein catheter for maintenance of anesthesia levels during all imaging procedures. Depth of anesthesia, hemodynamics, and body temperature were monitored every 15 min, as described during the surgical intervention. The body temperature was maintained above 37 °C during imaging procedures. Prior to PET/CT imaging, jugular vein access was established via percutaneous puncture and placement of a 4-F polyethylene catheter (Cook Medical, Bloomington, IN), which enabled venous administration of ^18^F-FDG for PET imaging. Prior to each PET scan, approximately 1 ml of venous blood was sampled to measure whole-blood glucose concentration using a commercially available blood glucose monitor (AlphaTRAK 2; Zoetis Inc., NJ, USA).

^18^F-FDG PET/CT imaging was performed 2 weeks following angioplasty of the right femoral artery using a hybrid PET/CT system (Discovery 690, GE Healthcare, Chicago, IL) 75 min after intravenous administration of ^18^F-FDG (dose 306.4 ± 38.3 MBq). The PET imaging field-of-view was set from the lower abdomen to the lower legs to ensure full scan coverage of the peripheral arteries of interest. CT images were first acquired with a slice thickness of 3.27 mm, at 120 kVp, and 300 mA, for image registration, attenuation correction, and manual segmentation of the femoral arteries. PET imaging was then performed for 8 min per bed position. PET data were reconstructed at 3.27 mm slice thickness with a pixel size of 2.73 mm using the OSEM algorithm with three iterations and 32 subsets, and Gaussian post-reconstruction filter with full width at half maximum of 4 mm. Following completion of hybrid PET/CT imaging, CT angiography images were acquired with iodinated contrast to facilitate identification of the femoral arteries and aid in PET image segmentation. CT angiography images were acquired at a slice thickness of 0.625 mm, at 300 mA, and 140 kVp. Intravenous contrast injections were performed using an integrated power injector (Stellant D, Medrad Inc, Warrendale, PA) at a constant rate of 3 ml/s and total volume of 30 ml, followed by a 0.9% saline flush at 3 ml/s.

### ^18^F-FDG PET/CT Image Analysis

All PET/CT image analysis was performed using commercially available software (PMOD Technologies LLC, Fallanden, Switzerland). Co-registered PET and CT images were used to define arterial regions of interest (ROIs). CT angiograms were utilized to aid in the identification and segmentation of the femoral artery for each animal. ROIs were then translated and resampled to match PET spatial resolution (3.27 mm) and quantify ^18^F-FDG uptake for the injured and control arteries on fused PET/CT attenuation images. For the arterial segmentation process, circular ROIs were used for each axial slice that were ~ 2 mm larger than the outer diameter of the femoral artery to account for the inherent limitations of PET spatial resolution and potential partial volume effects. Additionally, 2 human analysts performed PET/CT image segmentation of the femoral artery to evaluate inter-rater agreement of the PET image quantification process in the pig model. Given that the right hindlimb served as the injured limb in all animals for model consistency, image analysts were not blinded as to which femoral artery represented the injured or control vessel.

^18^F-FDG uptake values were quantified within each arterial ROI and expressed as standardized uptake values (SUVs). To obtain the average maximum SUV (i.e., mean SUV_max_) for both femoral arteries, maximum SUVs measured per ROI of each slice of the femoral artery were summed and averaged. ^18^F-FDG uptake for each femoral artery was ultimately evaluated and expressed as mean SUV_max_.

To measure the arterial diameters of native (control) and injured femoral arteries, CT angiography images were utilized and processed with FDA-approved DICOM viewer software (Osirix, Pixmeo, Switzerland).

### Vascular Tissue Collection and Analysis

Immediately following completion of PET/CT imaging at 2 weeks post-angioplasty, animals were euthanized and femoral arteries from the injured (right) and control (left) limbs were harvested, flushed, and washed with PBS to remove potential blood clots, and cut into 5 mm sections. Three 5 mm arterial samples were used to represent the injured portion of artery and three 5 mm samples from the corresponding anatomical location were used to represent the contralateral non-injured femoral artery. Samples were transferred to PBS-filled tubes to measure ^18^F-FDG radioactivity/uptake for each 5 mm section, which was expressed as percent injected dose per gram of tissue. All samples were weighed, counted, and corrected for radioactive decay using a gamma well counter (WIZARD2, PerkinElmer, Waltham, MA). One animal’s tissue samples were unable to undergo gamma counting due to mechanical failure of the gamma counter on the study date. Therefore, all analysis involving gamma counting included 7, not 8, datasets. Following completion of gamma counting, samples were fixed with 4% paraformaldehyde overnight and then stored in PBS at 4 °C until paraffin embedding. Embedded tissue was then sectioned at 4 μm thickness.

Hematoxylin and eosin (H&E) staining was performed on formalin-fixed paraffin-embedded arterial sections that were deparaffinized using xylene and grades of ethanol. Hematoxylin solution (Sigma Aldrich, GHS332) and Eosin Y (Sigma Aldrich, HT110332) were used according to the manufacturer’s protocol. For immunohistochemical staining (IHC) of CD64 and α-smooth muscle actin (α-SMA), embedded arterial sections were deparaffinized using xylene and grades of ethanol followed by antigen retrieval with citrate-based antigen unmasking solution (H-3300, Vector laboratories). After antigen retrieval, tissue sections were incubated with 3% H_2_O_2_ diluted in water at room temperature to quench endogenous peroxidase activity and blocked with 3% normal goat serum (Abcam, ab7481) in Background Sniper (Biocare Medical, LLC) for 30 minutes at room temperature to avoid nonspecific binding. Endogenous biotin was blocked using an avidin/biotin blocking kit (Abcam, ab64212) with avidin incubation for 15 min followed by biotin incubation for 15 min per the manufacturer’s protocol. Sections were then incubated with mouse anti-CD64 (1:1000, Abcam, ab140779) and mouse anti-α-Smooth Muscle Actin (anti-α-SMA) (1:1000, Abcam, ab7817) overnight at 4 °C. The next day, sections were incubated in biotinylated goat anti-mouse IgG secondary antibody for 30 min (Abcam, ab6788), followed by incubation in Vectastain Elite ABC-HRP Reagent, Peroxidase (PK-7100, Vector Laboratories) for 30 min. Sections were washed then visualized using the SignalStain DAB Substrate Kit (8059, Cell Signaling Technology).

Image quantification was performed using ImageJ (National Institutes of Health, Bethesda, MD) on images acquired at 20× magnification using 3 sections per artery (Axio Observer, Carl Zeiss, White Plains, NY). A plug-in on ImageJ function named “Local Thickness” was used to quantify vessel thickness [13]. Briefly, images of arteries, including the intima, media, and adventitia, were binarized. Then, a Euclidean distance transform algorithm was applied to create distance maps at every pixel contained by the vessels. The algorithm then computed all local thickness values as determined by the diameter of the largest circle at any point within the vessel region and provided a histogram of thickness values with a default total bin number of 256. The mean of all values was reported as the mean thickness. To quantify the total positively stained area of blood vessels, IHC images underwent color deconvolution to extract DAB-only images using ImageJ. All images were thresholded using a value determined from a positive control image. The threshold value was a mean of values determined by three different users.

One animal had complete arterial dissection in one of three samples, rendering image quantification for the section imprecise. Therefore, in one animal, two representative sections instead of three were used for gamma counting/histological evaluation as a quality-control measure to ensure consistency across all measures. H&E images for all animals were evaluated for total vessel thickness, while CD64 and α-SMA expression was reported as total positively stained area.

### Statistical Analysis

A Wilcoxon signed-rank test was used to compare differences in ^18^F-FDG uptake between injured and control femoral arteries from the in vivo PET/CT imaging and ex vivo gamma counting measures. Pearson’s correlation was used to assess the linear relationship between in vivo PET/CT imaging and ex vivo gamma counting measures of ^18^F-FDG uptake. To account for repeated measures within animals (three sections per artery per animal) linear regression model with mixed effects was used to compare differences in arterial thickness, α-SMA positive area, and CD64 positive area between control and injured femoral arteries. Animal ID was included as a random effect to account for within animal clustering. The associations between arterial uptake of ^18^F-FDG and (1) arterial thickness, (2) α-SMA positive area, and (3) CD64 percent area were assessed using a mixed-effects linear regression model with random effect for animal. Normalized residuals from the models were examined to assess the potential influence of observations. A paired *t*-test was used to compare differences in CT angiography-derived measures of arterial diameter between control and injured femoral arteries. Intra-class correlation coefficient was used to evaluate the inter-rater agreement for PET image quantification. A *p* value < 0.05 was considered statistically significant. All statistical analyses were performed using GraphPad Prism, version 10.1.0 for windows and R statistical software (v4.5.0; R Core Team 2025).

## Results

### In Vivo ^18^F-FDG PET/CT Imaging of Vascular Remodeling

In vivo PET/CT imaging 2 weeks after balloon angioplasty demonstrated qualitative increases in femoral artery uptake of ^18^F-FDG in injured versus control arteries (Fig. 1B–D). Quantitative PET image analysis, expressed as the mean SUV_max_ across the length of ballooned arterial segment, confirmed a significant difference for in vivo ^18^F-FDG uptake between injured and control femoral arteries (injured artery: 1.2 ± 0.7 SUV_max_; control artery: 0.8 ± 0.4 SUV_max_; *p* = 0.008) (Fig. 1E). Inter-rater agreement across 2 human analysts for quantification of femoral artery ^18^F-FDG uptake (i.e., mean SUV_max_) was good to excellent with an intraclass correlation coefficient of 0.88.

In vivo CT angiography imaging demonstrated a significant difference in femoral artery diameter at 2 weeks after balloon angioplasty between injured and control femoral arteries (injured artery: 5.2 ± 0.8 mm; control artery: 4.6 ± 0.6 mm; *p* = 0.04) (Table 1).

### Ex vivo Gamma Counting of Vascular ^18^F-FDG Uptake

**Fig. 2:**
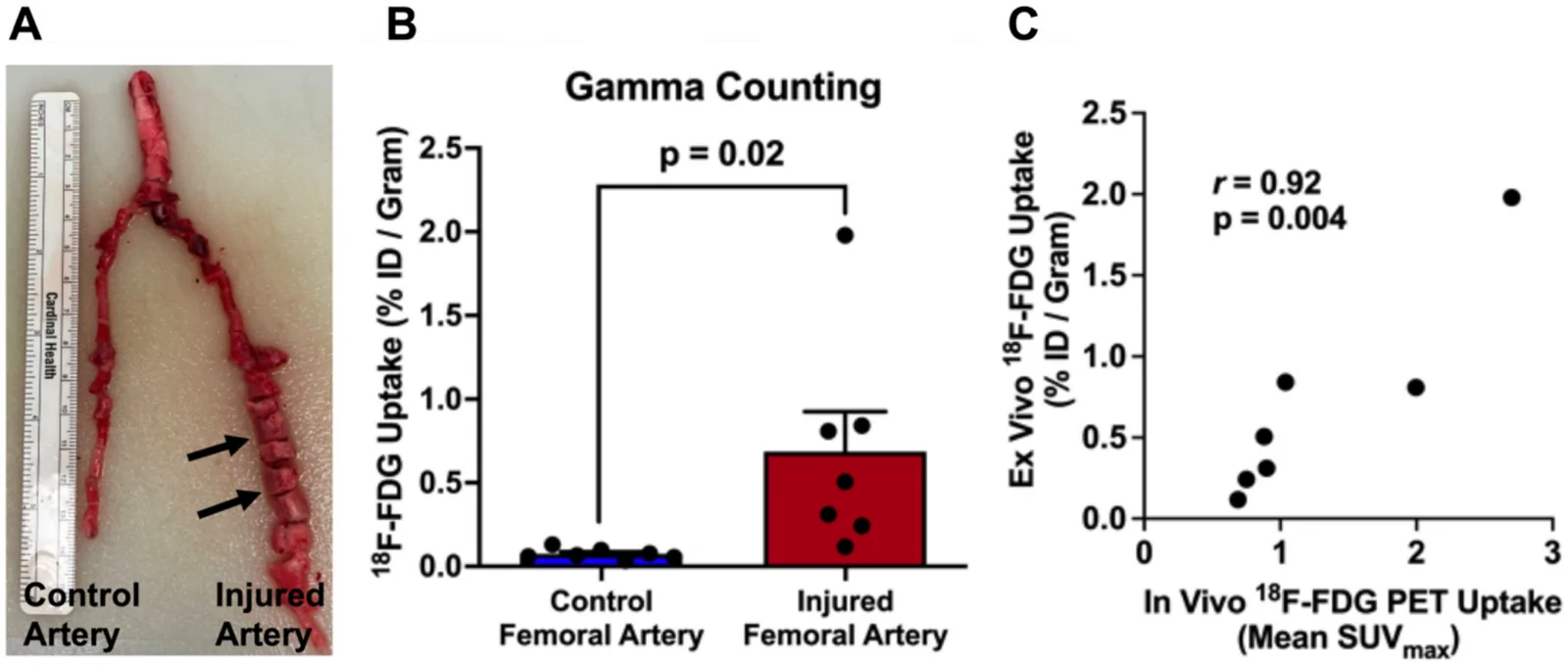
Ex vivo quantification and validation of ^18^F-FDG retention in angioplasty-induced injury of the femoral artery. **A** Representative example of excised peripheral arteries harvested at 2 weeks after balloon-induced injury for gamma counting and anatomically matched histological analyses (arrows denote region of injury). **B** Post-mortem gamma counting of excised arterial segments from injured and control femoral arteries demonstrated significantly higher ^18^F-FDG uptake in injured arteries 2 weeks after balloon angioplasty. **C** Pearson’s correlation demonstrated a significant association between quantitative analysis of ^18^F-FDG uptake from in vivo PET/CT imaging and ex vivo gamma counting, *n* = 7 animals. Data are shown as means ± SEM

Postmortem gamma counting of arterial samples at 2 weeks after balloon angioplasty demonstrated a significant increase in absolute measures of ^18^F-FDG uptake in injured versus control arteries (injured artery: 0.7 ± 0.6% injected dose/gram; control artery: 0.07 ± 0.03% injected dose/gram; *p* = 0.02) (Fig. 2B). Additionally, arterial uptake of ^18^F-FDG measured by ex vivo gamma counting was significantly and positively correlated with ^18^F-FDG uptake measured by in vivo PET/CT imaging (*r* = 0.92, *p* = 0.004) (Fig. 2C).

### Histological Analysis of Vascular Remodeling

**Fig. 3:**
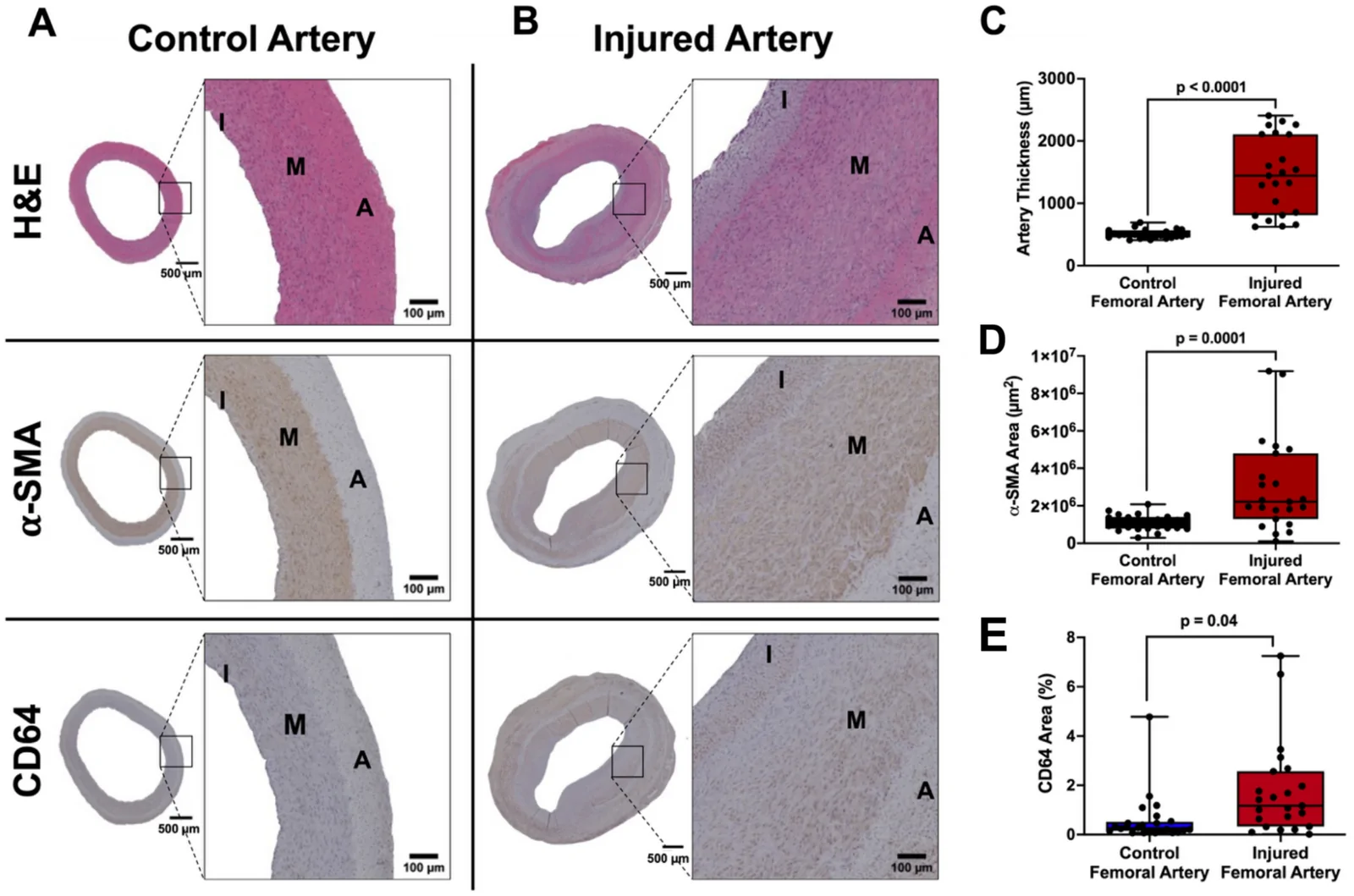
Histological evaluation of angioplasty-induced vascular remodeling. Representative H&E-, α-SMA-, and CD64-stained images of **A** non-injured control and **B** injured femoral artery sections revealed qualitative differences in vascular morphology, vascular smooth muscle cell content, and macrophage infiltration 2 weeks after balloon angioplasty. Quantitative image analysis further confirmed **C** a significant increase in arterial thickness, **D** α-SMA-positive area, and **E** CD64-percent positive area between control and injured femoral arteries. Lines in box plot represent medians, lower and upper box limits represent 25^th^ and 75^th^ percentiles, and whiskers extend to the minimum and maximum values, *n* = 8 animals. I intima, M media, and A adventitia

H&E, α-SMA, and CD64 staining of arterial sections demonstrated qualitative differences in arterial thickness and SMC proliferation between control and injured arteries 2 weeks after balloon angioplasty (Fig. 3A, B). Quantitative image analysis further confirmed significant increases in arterial thickness (injured artery: 1454.0 ± 615.3 μm; control artery: 505.8 ± 72.3 μm; *β* = 951.0; *p* < 0.0001), α-SMA positive area (injured artery: 3005049.9 ± 2457929.5 μm^2^; control artery: 1105610.3 ± 429915.0 μm^2^; *β* = 1909473; *p* = 0.0001), and CD64 percent positive area (injured artery: 1.8 ± 1.9%; control artery: 0.6 ± 1.0%; *β* = 1.2; *p* = 0.04) in injured versus control arteries (Fig. 3C–E).

### Association Between ^18^F-FDG Uptake and Markers of Remodeling

**Fig. 4:**
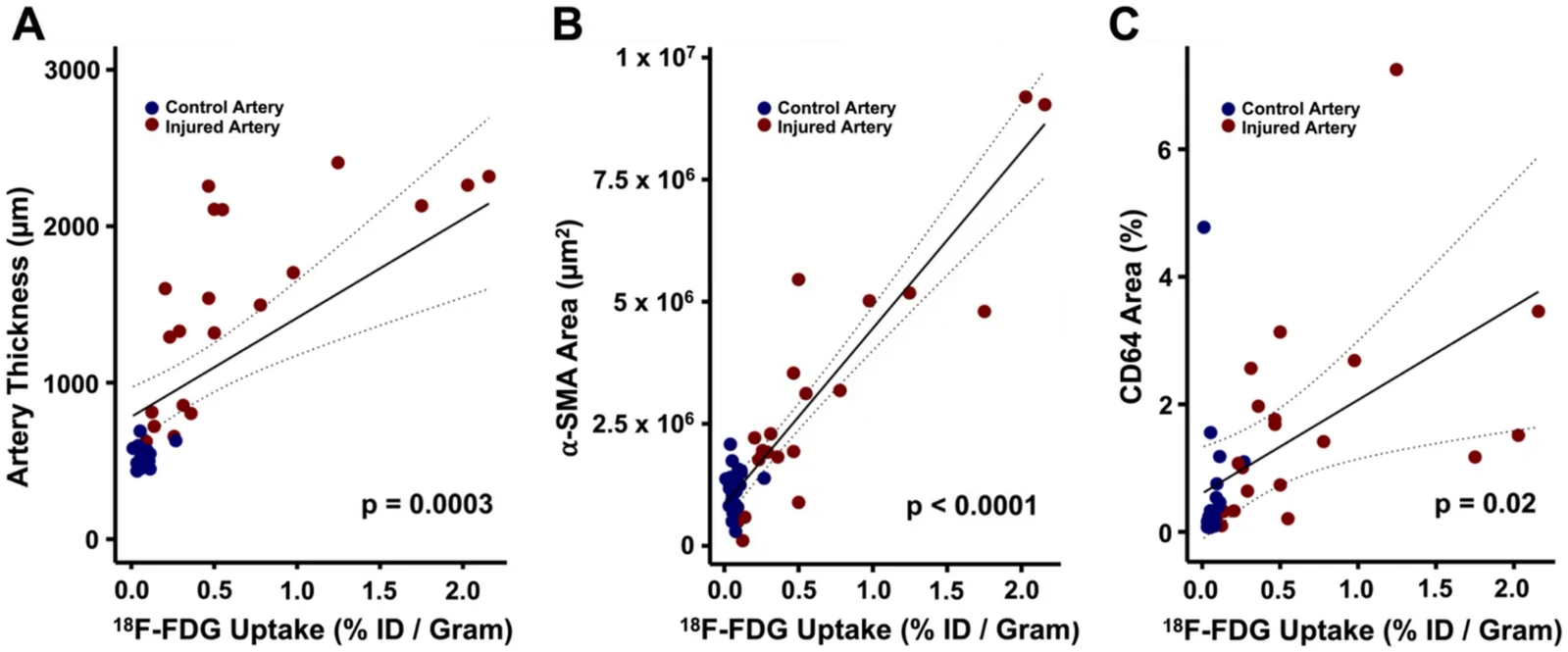
Association between vascular uptake of ^18^F-FDG and angioplasty-induced vascular remodeling. Linear regression analyses demonstrated significant positive associations between arterial uptake of ^18^F-FDG and **A** arterial thickness, **B** α-SMA-positive area, and **C** CD64-percent positive area in control and injured femoral arteries, *n* = 7 animals

Evaluation of the potential associations between vascular uptake of ^18^F-FDG in injured arteries (measured by gamma counting) and vascular remodeling characteristics demonstrated that ^18^F-FDG uptake was significantly and positively associated with artery thickness (*β* = 632.1; 95% CI 324.0, 940.2; *p* = 0.0003) (Fig. 4A), smooth muscle cell content (*β* = 3617371; 95% CI 2993449.1, 4241291.2; *p* < 0.0001) (Fig. 4B), and CD64 expression (*β* = 1.46; 95% CI 0.3, 2.7; *p* = 0.02) (Fig. 4C) at 2 weeks after balloon angioplasty.

## Discussion

The present study contributes to the existing vascular imaging and remodeling literature by providing unique insights into the relationship between markers of vascular remodeling and ^18^F-FDG retention in response to angioplasty-induced injury. Specifically, this study demonstrated that PET/CT imaging detects significant in vivo increases in vascular retention of ^18^F-FDG after angioplasty-induced injury in a large animal model, which was validated by ex vivo gamma counting of vascular samples. Further, histological analyses revealed that markers of vascular inflammation (i.e., macrophage expression) and remodeling (i.e., smooth muscle cell content, vessel thickness) were significantly associated with ^18^F-FDG retention in injured arteries. Collectively, the findings of this study suggest that ^18^F-FDG PET/CT imaging can non-invasively quantify unique aspects of vascular remodeling associated with angioplasty-induced injury.

Prior studies have shown an association between arterial retention of ^18^F-FDG and inflammatory markers, including macrophage expression, in the setting of clinical and pre-clinical atherosclerosis [14–16]. The findings of the present study agree with these prior studies by demonstrating a significant association between macrophage expression and ^18^F-FDG retention at 2 weeks after induction of injury in the porcine model. Although we found a significant association between ^18^F-FDG retention and macrophage expression, the association between ^18^F-FDG retention and smooth muscle content was stronger in our angioplasty model. This finding is corroborated by a prior pre-clinical atherosclerosis study that showed arterial ^18^F-FDG uptake on PET imaging was more closely linked to smooth muscle cell presence than macrophage expression [17]. While other atherosclerosis-focused studies have also evaluated the connection between ^18^F-FDG retention and the presence and metabolic activity of smooth muscle cells [10, 11], the present study is the first to evaluate the association between ^18^F-FDG retention, smooth muscle cell content, and vessel thickness in a large animal model of angioplasty-induced vascular injury. Our findings support the atherosclerosis-focused work of others that has found significant associations between ^18^F-FDG retention and smooth muscle cell presence; however, the additional finding in the present study demonstrating a direct link between ^18^F-FDG retention and injury-induced changes in vessel thickness is particularly unique. These findings collectively indicate that ^18^F-FDG PET/CT imaging may offer a novel in vivo paradigm for non-invasively monitoring vascular remodeling consequences of endovascular devices and could potentially serve as a unique approach for pre-clinical testing the efficacy of emerging PAD devices, such as drug-coated balloons, stents, and bioresorbable scaffolds. Given that ^18^F-FDG is a readily available, FDA-approved radioisotope, this PET/CT imaging approach could also be rapidly translated to PAD patients undergoing peripheral interventions to assess the prognostic value of ^18^F-FDG retention for predicting risk of post-intervention restenosis.

Although the present study validated ^18^F-FDG as a biomarker of angioplasty-induced vascular remodeling and the porcine model represented an ideal size for implementing clinically relevant endovascular devices, the animal model utilized in the present study was a healthy, young, non-atherosclerotic model. Therefore, future testing of this imaging approach in a more clinically relevant atherosclerotic animal model is warranted. Additionally, although we chose the 2 week PET/CT imaging time point in hopes of capturing both an early inflammatory and remodeling response to balloon angioplasty, 2 weeks after intervention proved to be relatively late for capturing a robust inflammatory response and the smooth muscle cell proliferative phase to injury that preceded the structural remodeling response. To evaluate the prognostic value of ^18^F-FDG PET/CT imaging for predicting the degree of vascular remodeling, implementation of earlier timepoint PET/CT imaging with longer-term follow-up after angioplasty would also be warranted. We also acknowledge that precise quantification of PET data in relatively small arteries can be challenging due to the inherent limitations of PET spatial resolution and potential partial volume effects when evaluating small objects; however, we believe our standardization and oversizing of arterial ROIs beyond the outer diameter of the femoral artery in addition to the measurement of PET data for control and injured limbs within the same animals helped offset any potential impact of partial volume effects in the present study.

## Data Availability

Data are available from the authors upon reasonable request.

## References

[1] Criqui, M. H., K. Matsushita, V. Aboyans, C. N. Hess, C. W. Hicks, T. W. Kwan, M. M. McDermott, S. Misra, F. Ujueta, American Heart Association Council on Epidemiology and Prevention, Council on Arteriosclerosis, Thrombosis and Vascular Biology, Council on Cardiovascular Radiology and Intervention, Council on Lifestyle and Cardiometabolic Health, Council on Peripheral Vascular Disease, Stroke Council. Lower extremity peripheral artery disease: contemporary epidemiology, management gaps, and future directions: a scientific statement from the American Heart Association. *Circulation*. 144:e171–e191, 2021. 10.1161/CIR.0000000000001005. PMC9847212 34315230

[2] Misra, S., M. H. Shishehbor, E. A. Takahashi, H. D. Aronow, L. P. Brewster, M. C. Bunte, E. S. H. Kim, J. R. Lindner, K. Rich, American Heart Association Council on Peripheral Vascular Disease, Council on Clinical Cardiology, Council on Cardiovascular and Stroke Nursing. Perfusion assessment in critical limb ischemia: principles for understanding and the development of evidence and evaluation of devices: a scientific statement from the American Heart Association. *Circulation*. 140:e657–e672, 2019. 10.1161/CIR.0000000000000708. PMC7372288 31401843

[3] Guez, D., D. R. Hansberry, C. F. Gonsalves, D. J. Eschelman, L. Parker, V. M. Rao, and D. C. Levin. Recent trends in endovascular and surgical treatment of peripheral arterial disease in the Medicare population. *AJR Am. J. Roentgenol.* 214:962–966, 2020. 10.2214/AJR.19.21967. 32097027

[4] Dun, C., D. P. Stonko, S. Bose, A. C. Keegan, K. M. McDermott, K. C. Rumalla, J. H. Black 3rd., C. A. Kalbaugh, M. A. Makary, and C. W. Hicks. Trends and factors associated with peripheral vascular interventions for the treatment of claudication from 2011 to 2022: a national Medicare cohort study. *J. Am. Heart Assoc.*13:e033463, 2024. 10.1161/JAHA.123.033463. PMC11292772 38958132

[5] Goodney, P. P., A. W. Beck, J. Nagle, H. G. Welch, and R. M. Zwolak. National trends in lower extremity bypass surgery, endovascular interventions, and major amputations. *J. Vasc. Surg.* 50:54–60, 2009. 10.1016/j.jvs.2009.01.035. 19481407

[6] Beckman, J. A., Schneider, and M. S. Conte. Advances in revascularization for peripheral artery disease: revascularization in PAD. *Circ. Res.* 128:1885–1912, 2021. 10.1161/CIRCRESAHA.121.318261. 34110904

[7] Gornik, H. L., et al. 2024 ACC/AHA/AACVPR/APMA/ABC/SCAI/SVM/SVN/SVS/SIR/VESS Guideline for the management of lower extremity peripheral artery disease: a report of the American College of Cardiology/American Heart Association joint committee on clinical practice guidelines. *Circulation*. 149:e1313–e1410, 2024. 10.1161/CIR.0000000000001251. PMC12782132 38743805

[8] Stacy, M. R. Radionuclide imaging of atherothrombotic diseases. *Curr. Cardiovasc. Imaging Rep.* 12:17, 2019. 10.1007/s12410-019-9491-7. PMC6561494 31191793

[9] Stacy, M. R. Molecular imaging of lower extremity peripheral arterial disease: an emerging field in nuclear medicine. *Front. Med.*8:793975, 2022. 10.3389/fmed.2021.793975 PMC8789656 35096884

[10] Pahk, K., C. Joung, S.-M. Jung, H. Y. Song, J. Y. Park, J. W. Byun, Y.-S. Lee, J. C. Paeng, C. Kim, S. Kim, and W.-K. Kim. Visualization of synthetic vascular smooth muscle cells in atherosclerotic carotid rat arteries by F-18 FDG PET. *Sci. Rep.* 7:6989, 2017. 10.1038/s41598-017-07073-3. PMC5539104 28765576

[11] Nogales, P., C. Velasco, L. Gonzalez-Cintado, D. Sharysh, A. Mota-Cobian, R. Izquierdo-Serrano, C. Torroja, D. Del Rio-Aledo, D. Morales-Cano, R. A. Mota, A. Benguria, A. Dopazo, F. Sanchez-Cabo, J. Vazquez, S. Espana, L. Carramolino, J. Mateo, and J. F. Bentzon. Atherosclerotic disease activity is associated with glycolytic enzyme expression across multiple cell types and is trackable by FDG-PET. *Sci. Transl. Med.* 17:eado6467, 2025. 10.1126/scitranslmed.ado6467. 40802740

[12] Chowdhury, M. M., J. M. Tarkin, M. S. Albaghdadi, N. R. Evans, E. P. V. Le, T. B. Berrett, U. Sadat, F. R. Joshi, E. A. Warburton, J. R. Buscombe, P. D. Hayes, M. R. Dweck, D. E. Newby, J. H. F. Rudd, and P. A. Coughlin. Vascular positron emission tomography and restenosis in symptomatic peripheral arterial disease: a prospective clinical study. *JACC Cardiovasc. Imaging*. 13:1008–1017, 2020. 10.1016/j.jcmg.2019.03.031. PMC7136751 31202739

[13] Dougherty, R., and K.-H. Kunzelmann. Computing local thickness of 3D structures with ImageJ. *Microsc. Microanal.* 13:1678–1679, 2007. 10.1017/S1431927607074430.

[14] Cocker, M. S., J. D. Spence, R. Hammond, R. A. DeKemp, C. Lum, G. Wells, J. Bernick, A. Hill, S. Nagpal, G. Stotts, M. Alturkustani, A. Adeeko, Y. Yerofeyeva, K. Rayner, J. Peterson, A. R. Khan, A. C. Naidas, L. Garrard, M. J. Yaffe, E. Leung, F. S. Prato, J.-C. Tardif, R. S. B. Beanlands, and C. A. I. Network. [18F]-fluorodeoxyglucose PET/CT imaging as a marker of carotid plaque inflammation: comparison to immunohistology and relationship to acuity of events. *Int. J. Cardiol.* 271:378–386, 2018. 10.1016/j.ijcard.2018.05.057. 30007487

[15] Tawakol, A., R. Q. Migrino, G. G. Bashian, S. Bedri, D. Vermylen, R. C. Cury, D. Yates, G. M. LaMuraglia, K. Furie, S. Houser, H. Gewirtz, J. E. Muller, T. J. Brady, and A. J. Fischman. In vivo 18F-fluorodeoxyglucose positron emission tomography imaging provides a noninvasive measure of carotid plaque inflammation in patients. *J. Am. Coll. Cardiol.* 48:1818–1824, 2006. 10.1016/j.jacc.2006.05.076. 17084256

[16] Zhang, Z., J. Machac, G. Helft, S. G. Worthley, C. Tang, A. G. Zaman, O. J. Rodriguez, M. S. Buchsbaum, V. Fuster, and J. J. Badimon. Non-invasive imaging of atherosclerotic plaque macrophage in a rabbit model with F-18 FDG PET: a histological correlation. *BMC Nucl. Med.* 6:3, 2006. 10.1186/1471-2385-6-3. PMC1479805 16725052

[17] Al-Masshadi, R. H., L. P. Tolbod, L. O. Bloch, M. M. Bjorklund, Z. P. Nasr, Z. Al-Mashhadi, M. Winterdahl, J. Frokiaer, E. Falk, and J. F. Bentzon. 18Fuorodeoxyglucose accumulation in arterial tissues determined by PET signal analysis. *J. Am. Coll. Cardiol.* 74:1220–1232, 2019. 10.1016/j.jacc.2019.06.057. 31466620

